# The Impacts of Information-Sharing Mechanisms on Spatial Market Formation Based on Agent-Based Modeling

**DOI:** 10.1371/journal.pone.0058270

**Published:** 2013-03-06

**Authors:** Qianqian Li, Tao Yang, Erbo Zhao, Xing’ang Xia, Zhangang Han

**Affiliations:** Department of Systems Science, Beijing Normal University, Beijing, P. R. China; Cinvestav-Merida, Mexico

## Abstract

There has been an increasing interest in the geographic aspects of economic development, exemplified by P. Krugman’s logical analysis. We show in this paper that the geographic aspects of economic development can be modeled using multi-agent systems that incorporate multiple underlying factors. The extent of information sharing is assumed to be a driving force that leads to economic geographic heterogeneity across locations without geographic advantages or disadvantages. We propose an agent-based market model that considers a spectrum of different information-sharing mechanisms: no information sharing, information sharing among friends and pheromone-like information sharing. Finally, we build a unified model that accommodates all three of these information-sharing mechanisms based on the number of friends who can share information. We find that the no information-sharing model does not yield large economic zones, and more information sharing can give rise to a power-law distribution of market size that corresponds to the stylized fact of city size and firm size distributions. The simulations show that this model is robust. This paper provides an alternative approach to studying economic geographic development, and this model could be used as a test bed to validate the detailed assumptions that regulate real economic agglomeration.

## Introduction

Researchers have become increasingly interested in the geographic aspects of economic development. P. Krugman [1] studied the role of geography in economic development and argued that the role of geography should have become a mainstream concern within economics long ago. Centripetal forces (such as forward and backward linkages in production and increasing returns in transportation) and centrifugal forces (such as factor immobility and land rents) can result in a process of self-organization in which otherwise similar locations end up playing notably different economic roles. Economic analysis has shown that centers may emerge as a result of attempts by producers to minimize the costs of production and delivery or because larger cities can support a wider range of activities [1].

There are studies emphasizing that physical geography is highly differentiated and that these differences have a large effect on economic development. In addition, some studies have concluded that economies benefit from specific geographic advantages such as coastlines and areas connected to the coast by navigable rivers, and these areas are more densely populated than the hinterlands [2]. The new economic geography, however, shows how increasing returns to scale, agglomeration economies, transport costs, and product differentiation can lead to a highly differentiated spatial organization of economic activity (including cities, hubs and spokes, international division of labor between industry and agriculture, and so on), even when the underlying physical geography is undifferentiated [1]. P. Krugman noted that small random historical events might have large consequences for economic geography [1]; for a review of the new economic geography, see references [3], [4]. The two approaches, which attempt to explain why the economic destinies of locations might diverge both with and without inherent advantages or disadvantages, are complementary, rather than contradictory [1].

The present study follows the new economic geography and considers only locations without geographic advantages or disadvantages that can influence economic development. As reviewed by B. Arthur in reference [5], due to historical path dependence and positive feedback, companies may start out equal but end with asymmetrical outcomes.

The primary question in economic geography could be expressed as follows. Many economic activities are concentrated geographically. Most people live in large, densely populated metropolitan areas. Many industries–including service industries such as banking–are also concentrated geographically, and such clusters are an important source of international specialization and trade. What causes this spatial clustering?

To address this question, P. Krugman provided a two-region analysis of the centripetal and centrifugal forces [1], and J. Thisse used a spatial integration of the resulting benefit of a firm obtained from another firm [3]. In contrast, we use agent-based computational experiments in which agents have a certain probability of meeting; when the interaction occurs, there may be a transaction between the corresponding agents [6]. This is, as far as we know, the first agent-based model in the economic geography field. This type of model has been viewed as potentially useful for studying the emergence of market towns where people meet to trade goods or exchange information [3].

We hypothesize that a key factor may be the information-sharing mechanism. The information “function” has been studied in several different areas. In many markets, the asymmetry in the available information between sellers and buyers tends to result in a reduction in the average quality of goods and also in the size of the market (the lemon principle) [7]. Reference [8] analyzed a binary game in which the players use a finite set of *ad hoc* strategies to make their decisions based on the historic record, and interesting patterns of cooperation and competition arise. The exchange of knowledge has been shown to increase the efficiency of the market [9]. The term “information sharing” in the present paper corresponds to the terms “market statistic” in reference [7], “past record” in reference [8] and “knowledge exchange” in reference [9]. K. Anand and H. Mendelson considered the information structure of a firm to consist of two components: *knowledge* that cannot be transferred between market areas and *data* that can be transferred [10]. It was shown that both too little and too much information sharing is sub-optimal for some systems [11], [12].

Regarding spatial structures, a famous pioneering work by R. Axtell *et al*. closely reproduces important spatial and demographic features of the Anasazi in the Long House Valley from about A.D. 800 to 1300 using a multi-agent computational model [13]. The rich paleoenvironmental record permits the computer to create a dynamic resource landscape that accurately replicates actual conditions in the valley, and quite accurate results were obtained. R. Axtell *et al*.’s work used actual measures of environmental variability to create a dynamic landscape of annual potential maize production that resulted in the spatial pattern, while the present study follows the idea that the spatial pattern may emerge from the homogeneous initial state that is mentioned in the second paragraph. Spatial integration and segregation are of interest to researchers thanks to T.C. Schelling’s analysis of the segregation in social environments [14]. He showed that even if people only have a very mild preference for living with neighbors of their own type, as they move to satisfy these preferences, complete segregation would occur. An agent-based modeling of global pattern formation and ethnic/cultural violence published in Science magazine showed that the characteristic size of the spatial clustering of different ethnic groups is the underlying cause for ethic violence [15]. A study by M.W. Macy and Y. Sato indicated that with moderate mobility, agents learn to read telltale signs of character, which enables them to take advantage of better opportunities outside of the neighborhood. Without an effective signaling system, a global market cannot emerge [16].

There are very few works that study the effects of different types of information-sharing mechanisms on the spatial structure of agent-based systems. It is thus necessary to systematically analyze the impact of a gradual change in information sharing on the spatial structure of the market. According to reference [17], information-sharing mechanisms can be separated into direct experience, witness information and sociological information. In this study, we examine three levels of information sharing: 1) no information sharing (in which agents utilize only personal information); 2) information sharing among agents within a community, as emulated by information sharing within a group of friends; and 3) information sharing among agents through environmental signals, which are analogous to pheromones in the biological world. The spectrum in the present study spans from no information sharing to a point just shy of global information sharing. This spectrum enables us to assess the extent to which the information-sharing mechanism alone contributes to the agglomeration of economic activities.

The agent-based model used in this paper has similarities and differences when compared to self-organizing maps (SOMs). T. Kohonen defined a self-organizing map as a sheet-like artificial neural network where neighboring cells compete in their activities by means of mutual lateral interactions and develop adaptively into specific detectors of different signal patterns [18]. Learning in this type of neural network is competitive, unsupervised and self-organizing. Both agent-based models and SOMs are discrete in time and space. The individual cell or agent can act independently during the simulation, and its learning is unsupervised, self-organized and stochastic. Cells and agents all adapt through interactions with others.

The critical difference between an agent-based model and an SOM is that an agent-based model introduces adaptive agents that move independently on the lattices. The locus on the lattice is not moving and can have its own attributes such as the cells in an SOM. Agents can have cognition features and hence are more representative when modeling social-economic systems [19].

## Model

In this model, we abstract the definition of the actual market to be a process in which information can influence the moving behavior of individual traders. Here, several details of real economic activities (markets) were simplified. Disregarding many practical factors, such as moving cost, trade demand diversity, market structure, information dissemination efficiency and the economic environment, we modeled the spatial evolution of the market as a result of changes in the trading positions of agents with different information-sharing mechanisms.

The extent of information sharing serves as an “order parameter”, a measure of the degree of order of collective behavior, to which the other aspects of behavior are coupled. The order parameter is a typical research paradigm in complexity research. For example, the local ethnic patch size contributes to the prediction of local violence in reference [20], and mobility contributes to the explanation of market structure in the US and Japan [16].

### 1 Agents and the Environment

We represent economic activity as the trade between two different types of economic agents.

In our model, there are *N* agents consisting of *N/*2 buyers and *N/*2 sellers. Initially, these agents are distributed randomly on a two-dimensional *L×L* lattice with periodic boundary conditions. Each agent can move independently, and the movement of the agents is influenced by the available information. When an agent moves out of the lattice on one side, it enters from the other side. Each locus in the lattice can be occupied by only one agent. Two agents can trade when the distance between them is within their sight range, which is defined below. The overall spatial structure of the market will evolve with different information-sharing mechanisms.

### 2 Agent Behavior

Each agent in the lattice exhibits three types of behavior: trading, processing information and moving. In each time-step, no agent can move more than once or trade more than once. Seller agents are chosen in a random order in each time-step, and for each seller, an available buyer (one that has not yet traded within this time-step) within sight range is selected. If more than one buyer is available within sight range, one of these is randomly selected. If no buyers are available within sight range, the seller waits until the next time-step. If a trade takes place, each trader (buyer and seller) either remembers the current trade position or leaves a “pheromone” on the current position depending on the information-sharing mechanism, which will be addressed later.

Afterwards, an agent, whether it has successfully selected a trading partner or not, will always try to move. We assume that each agent moves with probability *1-P* to a new position selected according to each of the three information-sharing mechanisms. Agents may also move to another site on the lattice with probability *P* due to exploration needs. This type of probabilistic path selection is frequently used in Ant Colony Optimization (ACO) [21]. The exploration probability also reflects bounded rationality when many factors (that we do not take into account in this paper) may influence the agent to take a less optimal site. If the chosen destination is occupied, the agent must randomly select another empty locus within its sight range. If there is no vacant locus, the agent waits until the next time-step.

We measure the time-dependent variables across time or the size of the market when the system reaches a dynamic equilibrium after the transient process.

### 3 Information-sharing Mechanisms

In this paper, we consider three information-sharing mechanisms: the use of personal information alone with no information sharing among agents, information sharing via pheromone-like signals and information sharing in communities maintained by each agent. The details are as follows.

#### 3.1 Pheromone information sharing

The sharing of information through pheromones generally describes a mode of communication in biological systems. Several representative studies [22] include thresholds in their models of pheromone-like communication, and this mechanism has been found to perform well in task allocation [23], [24]. Economic activity may result in the development of infrastructures and facilities [1], and similar to ant colony chemotactic features, these infrastructures and facilities can attract agglomerations of latecomers.

In our model, after a trade takes place, the buyer agent and the seller agent each leaves a pheromone in the amount of *H_0_* at the site of the trade. During each time-step, each agent identifies the site within its sight range where the amount of pheromone is the largest and jumps to that site with probability *1-P*. If another agent occupies that site, the agent randomly selects another site within its sight range. If no vacancy is available, the agent waits until the next step.

The pheromone evaporation rate is a commonly used concept in ACO. The amount of a pheromone, *H*, will become *H(1−α)^n^* after *n* time-steps with *α* representing the evaporation rate. In this case, the agents neither communicate with each other nor use their trading memory. The only available information is provided by the pheromones. This situation represents localized global information sharing. The information is *global* because all agents are allowed to obtain access to the information and *localized* because only those agents that come close to a site can access that information.

#### 3.2 No information sharing

When agents utilize only personal information, they conduct economic activities based on local information alone without using any information that may be transferred from other agents. This situation corresponds to the fourth coordination structure, *no information,* in reference [10]. In this mechanism, each individual cannot access any information other than its own previous experience.

When a trade takes place, each trader (buyer and seller) memorizes the location of this trade with a memory strength of *S_0_*. An evaporation rate *β* is introduced such that the intensity of memory *S* becomes *S(1-β)^n^* after *n* time-steps. During each time-step, the agent examines its own memory record to identify the locus within its sight range with the largest *S* value and jumps to that site with probability *1-P*. If that site is occupied, the agent moves randomly to another locus within its sight range. If there is no vacant locus, the agent waits until the next time-step.

#### 3.3 Friend information sharing

Agents in the real world might not isolate themselves, thus using only their own knowledge; however, they also might not have the ability to travel widely to obtain (localized) global information, which would waste significant time and energy. Real-world agents usually communicate and share information within a group of friends. Therefore, between the above-mentioned two extremes, we model a typical mid-level information-sharing mechanism in which the agents can share information in a community. This mechanism corresponds to the second (decentralized) coordination structure in reference [10], in which decisions are made separately in each market using local knowledge and data. In the present study, however, an agent shares information with a group of agents and the information is only a trade history memory.

Initially, each agent randomly chooses *k* friends of the same type. When the agent tries to move, it asks for suggestions from all of these friends. Each friend then suggests the best positions in its memory that are within the sight range of the asking agent. Considering both its friends’ suggestions and its own memory, the asking agent chooses the best site and jumps to that site with probability *1-P.* If the chosen site is occupied, the agent walks randomly. In this information-sharing mechanism, the agents retain their own memory but do not memorize their friends’ suggestions. If there is no vacant locus, the agent waits until the next time-step.

### 4 A Unified Model

We next attempt to identify a unified model that describes all three of the information-sharing mechanisms described above based on changes to the value of a specific parameter. The friend information-sharing mechanism is equivalent to the no information-sharing mechanism when the number of friends *k = *0; however, the friend information-sharing mechanism cannot be made equivalent to the pheromone information-sharing mechanism strictly by changing the value of *k*. In the friend information-sharing mechanism, each agent chooses the potential trading position that is most strongly represented in the memories of all of its friends that are within its sight range. This memory representation is different from the pheromone signal, which is composed of the sum of all personal memories. Thus, even when *k* is very large, we still need to sum up the suggestions within the sight range of the asking agent to be equivalent to the pheromone model.

We introduce a memory overlapping mechanism to unify the three information-sharing mechanisms; this mechanism is designed as follows:

1. The symbols
2. The symbols used in the unified model are listed in Table 1.
3. The trading rules

**Table 1:** The symbols used in the unified model.

Symbols	Description
*W*	The set of loci in the two-dimensional *L×L* lattice
*A*	The set of all agents (both buyers and sellers)
*B*	The set of buyers
*S*	The set of sellers
*ε_i_*	The Moore neighborhood of agent *i, i∈A, ε_i_∈W*
*F_i_*	The set of friends of agent *i*, *i∈A*
*M_ip_*	The memory of agent *i* about locus *p, i∈A, p∈W*
*M_0_*	The unit memory strength of an agent about the trading locus

Agent *i∈S* located at locus *p_0_∈W* follows three trading rules at each time-step:

  - Agent *i* trades with agent *j* when *j* has not traded with any other agent in the current time-step, where *j∈B* and *p_j_∈ε_i_*. Otherwise, *i* waits until the next time-step.
  - The memory of agent *i* about locus *p* is updated as follows: 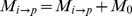, where 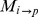 evaporates at rate *α*.
  - If there is an unoccupied position *p∈ε_i_* that satisfies,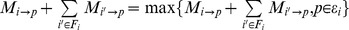, *i* jumps to that site with probability *1-P*. Otherwise, *i* commits a random walk to another *p∈ε_i_* with probability *P*.

In this unified model, each agent attempts to find a trader within its sight range. If a trader is available to the selecting agent, a trade takes place and *M_0_* is added to the memory of this agent for the trading locus. If no trader is available, the agent waits until the next time-step. After trading, each agent adds its friends’ memories and its own memory together to calculate the memory about each locus within its sight range. The locus with the largest resulting memory value is chosen by the agent as the next potential trading position. If this chosen locus is occupied, the agent jumps randomly within its sight range.

## Measurements

We analyze three statistics: the degree of clustering, simple social entropy and the successful trading ratio. The degree of clustering and simple social entropy measure the market spatial structure. The successful trading ratio measures the market efficiency.

In this study, we use the DBSCAN (density-based spatial clustering of applications with noise) algorithm, which was proposed by M. Ester [25], to distinguish clusters. DBSCAN is a widely adopted method to discover clusters of arbitrary shapes in spatial data. DBSCAN does not need to specify the number of clusters in the data a priori, as opposed to k-means; instead, DBSCAN can find arbitrarily shaped clusters, even a cluster surrounded by (but not connected to) a different cluster. A similar algorithm is proposed in reference [26]. The basic idea of DBSCAN is that for each point in a cluster, the neighborhood of a given radius *ε* must contain at least *MinPts* points, where ε and *MinPts* are input parameters. If the neighborhood of point *p* contains at least *MinPts* points, a cluster with *p* as the core point is created. All of the points that are density-reachable from the core point are subsequently retrieved and placed into the cluster. The algorithm terminates when no new point can be placed into any cluster.

### 1 Degree of Clustering

The formation of a spatially heterogeneous market is a process of agent congregation. It is therefore necessary to introduce a measurement of the degree of clustering, which is defined as follows:
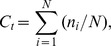
where *n_i_* is the number of agents within the sight range *r* of agent *i*. *C_t_* calculates the average number of agents in all neighborhoods at time-step *t*.

### 2 Simple Social Entropy

R. Gorelick and S. Bertram studied various indices that measure diversity in a system [27]. They categorized the measurements into four families: Shannon’s index/entropy, Simpson’s index, geometric mean, and standard/absolute deviation. Shannon prescribed three properties for a measure of information uncertainty: continuous, monotonic and recursive. T. Balch noted that Meyer’s metric 
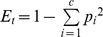
 (Simpon’s index in reference [27]) is continuous and monotonic; however, it is not recursive [28]. Shannon’s information entropy (called simple social entropy in [28]), however, meets all three criteria. With this benefit and for simplicity, the current study uses simple social entropy to measure social diversity, which can describe the order of spatial agglomeration in multi-agent systems. If all agents can be categorized (using DBSCAN) into *c* clusters, we set *P_i_ = N_i_/N,* where *N_i_* is the number of agents in cluster *i*, *N* is the total number of agents in the system, and *i = *1, 2, 3, …, *c*. Simple social entropy is defined as Shannon entropy.
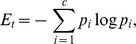
where *E_t_* is the simple social entropy at time-step *t*.

### 3 Successful Trading Ratio

Market efficiency is typically measured by the total surplus of both suppliers and consumers. In our model, the trade process is simplified: price curves are not considered, and we set a unit benefit to a pair of traders for a successful trade. Thus, the market efficiency can be measured by the frequency of successful trades, which is defined as.
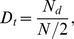
where *D_ t_, N_d_* and *N/*2 are the successful trading ratio, the number of successful trades and the maximum number of trades at time-step *t*. The larger the successful trading ratio is, the higher the market efficiency.

### 4 The Parameters of the Model

The amount of information produced by each trade transaction is set as a constant. The value of this constant does not affect the simulation results. A low pheromone evaporation rate means slow adaptation and a high evaporation rate means rapid adaptation. Studies have shown that with the evaporation rate ranging from 0.01 to 0.50, the expected quality of the optimization decreases rapidly (for a review, see reference [29]). Hence, we set the evaporation rate to *α = *0.01. Following is a sensitivity analysis of several parameters of the model.

#### 4.1 Population density and sight range

The population density, calculated as the number of agents *N* over the size of the lattice, has a considerable effect on the evolutionary results of most of the agent-based models. Obviously, the larger the value of *L*, the more representative the model is of the real market, and at the same time, the higher the computational complexity of the model. The sight range of the agents determines the level of information sharing between them. Taking both external validity and simplicity of computation into consideration, we set *L = *100 and compare the degree of agent agglomeration at different sight range values and at different densities by adjusting the value of *N*.

**Figure 1:**
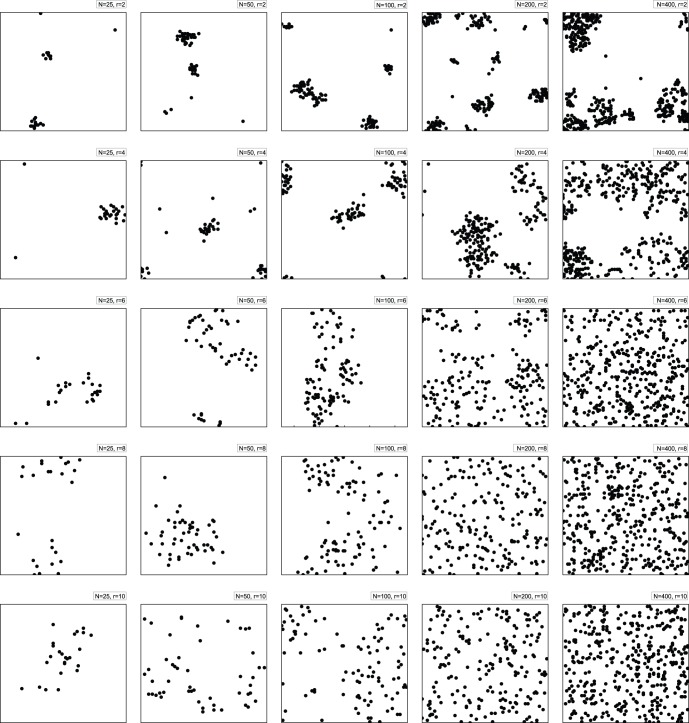
The spatial structure of the market for different numbers of agents and different sight ranges. This figure illustrates the spatial distribution of the agents for *k/N* = 0.99 on the two-dimensional *L×L* lattice when the simulation terminates at 100,000 time-steps, with the sight range *r* varying from 2 to 10 (top to bottom rows) and the number of agents varying from 25 to 400 (left to right columns). The sight range and the agent number values are marked on each plot. For *r = *2, the agglomeration of the agents is much more remarkable than for the other cases. As explained in the text, a larger sight range increases the random movements of agents throughout the lattice, which decreases the likelihood that agents will aggregate and form clusters. Even in the cases when *r = *2 and *r* = 4, where the agglomeration is relatively better than for the larger sight range cases, we can see that an increase in the number of agents (*N*≥200) is associated with a much more diffuse distribution of agents on the lattice. Therefore, setting a large number of agents does not ensure agglomeration. An increase in the number of agents also requires additional computational time. *N = *100 is therefore set to ensure agglomeration while reducing excessive computation.

Figure 1 simulates the evolution of the spatial distribution for the pheromone information-sharing mechanism (the number of friends over the total number of agents ratio *k/N* = 0.99, will be addressed later), with *r* varying from 2 to 10 (top to bottom rows) and the number of agents varying from 25 to 400 (left to right columns). Each plot is a spatial distribution of the agents on the two-dimensional *L×L* lattice when the simulation terminates at 100,000 time-steps.

For *r = *2, the agglomeration of the agents is much more remarkable than for the other cases. Because the Moore neighborhood of each agent contains *(*2*r+*1*)^2^−*1 grid cells, these neighborhoods are larger than 100 grid cells for *r>*5. In this case, a larger sight range means that the locus with the largest amount of pheromone can be identified by many more agents. In our model, each locus can be occupied by only one agent, and the other agents who find this locus must jump somewhere else randomly. A larger sight range increases the random movements of agents throughout the lattice, which decreases the likelihood that the agents aggregate and form clusters. Finally, we set *r = *2.

Even in the cases when *r = *2 and *r = *4 where the agglomeration is relatively better than it is in the larger sight range cases, we can see that an increase in the number of agents (*N*≥200) is associated with a much more diffuse distribution of agents on the lattice. Therefore, setting a large number of agents does not ensure agglomeration. An increase in the number of agents also requires additional computational time. Balancing between the effectiveness and the efficiency of the model, we set *N = *100.

#### 4.2 Cluster radius

The cluster radius *R_c_* reflects the scale of clustering on the lattice. On the one hand, too large a cluster radius, such as *R_c_*>5, makes it hard to distinguish between the aggregating state and the evenly random distribution because the distance between the core agent and the marginal agents is larger than *L/*2* = *50. Under this condition, all of the agents on the lattice can be placed in a single large cluster. On the other hand, too small of a cluster radius, such as *R_c_<*5, decreases the robustness of a cluster against random interruption. We set *R_c_* = 5. We will demonstrate in the Results section that the results obtained in this paper are robust to varying *R_c_* values.

#### 4.3 Random walk probability

**Figure 2:**
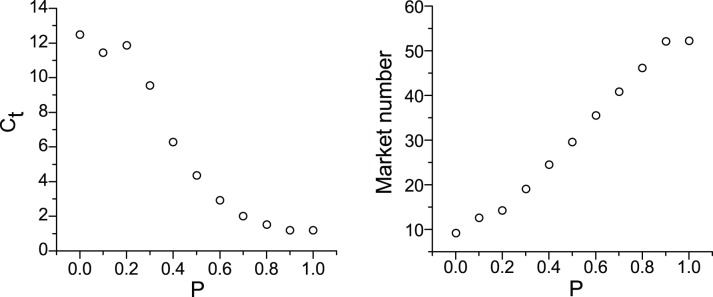
The effects of the random walk probability on the degree of clustering and the market number. This figure shows that the larger the value of *P*, the smaller the degree of clustering *C_t_* and the lower the market number. In this model, we set *P* = 0.10 because *P* = 0.10 leads the model to produce interesting results, i.e., clustering. The fact that agglomeration does not happen for other values is also a valid and meaningful result.

The random walk probability exhibits characteristics similar to those of temperature in a physical system. A large random walk probability increases the chance that an agent will choose another locus even when the best locus is not occupied. As a result, the agents in a cluster more readily jump out, which contributes to the collapse of the cluster. As Figure 2 shows, the larger the value of *P*, the smaller the degree of clustering and the lower the market number. In this model, we set *P* = 0.10, as *P* = 0.10 drives the model to produce interesting results, i.e., clustering. The fact that agglomeration does not occur for other values is also a valid and meaningful result.

#### 4.4 Number of friends

**Figure 3:**
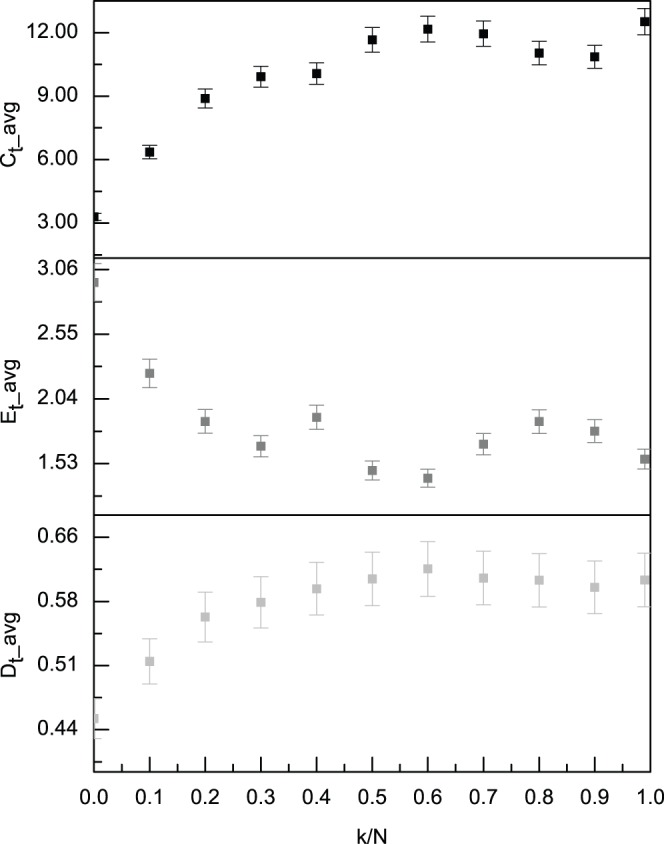
The friend number’s effects on the average successful trading ratio, market clustering degree and simple social entropy. With an increase in the number of friends per agent *k/N*, this model shifts from no information sharing to more information sharing. This figure shows that as *k/N* increases from 0 to 0.99, the averages of the successful trading ratio *D_t_* and the degree of clustering *C_t_* increase, and the average simple social entropy *E_t_* decreases. The error bars are also shown. All of the averages are calculated after the first 10,000 time-steps.

The number of friends, *k*, and hence the ratio *k/N*, is a key parameter in the unified model. Each agent shares more information with an increase in the number of friends per agent. Figure 3 shows that as *k* increases from 0 to 99, *k/N* increases from 0 to 0.99, the successful trading ratio and the degree of clustering increase, and the simple social entropy decreases. Having more friends provides agents with access to more information and enables more successful trades. In this case, clusters readily form, and the entire system becomes better organized with low simple social entropy.

In the unified model, we set *k/N = *0, *k/N = *0.10 and *k/N = *0.99 as three typical cases for the degree of information sharing, ranging from no information sharing to almost complete information sharing within the sight range. At *k/N* = 0, each agent has no information exchange with others. As the values of *k/N* range between 0.10 and 0.99, each agent has access to the superposition of the memories of all of its *k* friends plus its own memory about each locus. Specifically, at *k/N* = 0.99, each agent has access to the superposition of the memories of all of agents including its own.

The parameters of the model are listed in Table 2.

**Table 2:** The parameters of the model.

Description	Parameter	Value
Side length of the lattice	*L*	100
Total number of agents	*N*	100
Random walk probability	*P*	0.10
Sight range	*r*	2
Cluster radius	*R_c_*	5
Amount of information available after a trade	*M_0_*	100
Information evaporation rate	*α*	0.01
Number of friends in each information-sharing mechanism	*k*	0∼99

## Results

The simulation results for the various information-sharing degrees in the unified model are presented in this section. The termination criterion is set at 100,000 time-steps because the system becomes almost stationary at this time. All of the statistics are calculated after the first 10,000 time-steps.

### 1 Market Spatial Structure

**Figure 4:**
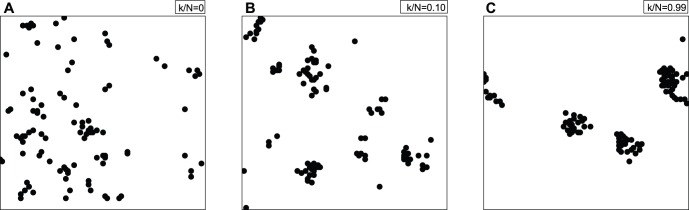
Market spatial structures resulting from the degree of information sharing. Each plot is a spatial distribution of the agents on the two-dimensional *L×L* lattice. Panel (A) with *k/N* = 0 represents no information sharing, panel (B) has *k/N* = 0.10 and panel (C) has *k/N* = 0.99. The agents scatter on the lattice without agglomeration in Panel (A). The distribution of the agents in Panel (B) appears to be less scattered than that in Panel (A), and several small clusters were observed. Only in Panel (C) can we observe the agglomeration of the agents into obvious clusters. This finding shows that the more information sharing there is, the more likely it is that economic centers will form.

The extent of information sharing has a profound impact on economic agglomeration. Each plot in Figure 4 is a spatial distribution of the agents on the two-dimensional *L×L* lattice. Panel (A) of Figure 4 shows the spatial structure of the market that results from no information sharing (*k/N* = 0). The agents scatter on the lattice without any hint of agglomeration. Panel (B) shows the spatial structure of the market that results from *k/N* = 0.10. The distribution of the agents appears less scattered than that in Panel (A), and several small clusters emerge. The highest level of information sharing *k/N* = 0.99, as Panel (C) shows, results in the agglomeration of the agents into obvious clusters. This finding shows that greater information sharing is likely to lead to the formation of economic centers.

### 2 Degree of Clustering

**Figure 5:**
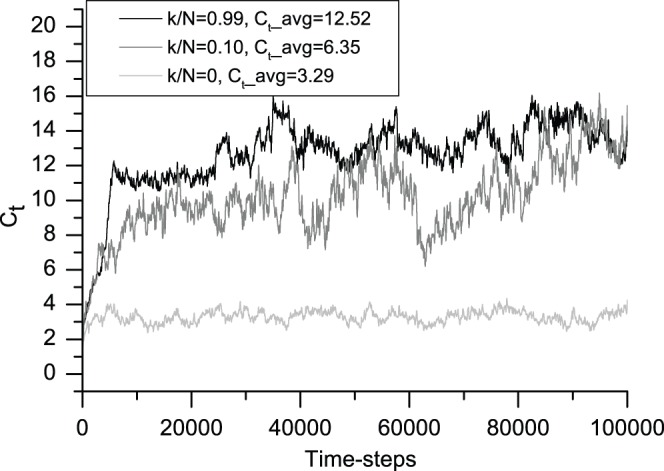
Evolution in the degree of clustering associated with the degree of information sharing. This figure shows the 200-iteration moving average of the degree of clustering. From bottom to top, light gray (*k/N* = 0), gray (*k/N* = 0.10) and black (*k/N* = 0.99) correspond to typical cases from no information sharing to the most information sharing, respectively. The average degrees of clustering *C_t_avg_* for the three cases are 3.29, 6.35 and 12.52, respectively. The agents in the pheromone-like mechanism show a much tighter integration than in the other two. All of the averages are calculated after the first 10,000 time-steps.

The degree of clustering reflects the degree of agglomeration in an economy. From the degree of clustering, we can see the average number of agents per neighborhood in a system. As the number of friends per agent increases, the degree of clustering clearly increases. In Figure 5, the top (black) curve denotes the change over time in the degree of clustering associated with the information-sharing amount *k/N* = 0.99, the middle (gray) curve denotes that associated with *k/N* = 0.10, and the bottom (light gray) curve denotes that associated with the no information-sharing mechanism. The results support the intuitive conclusion that information helps to promote clustering.

### 3 Simple Social Entropy

**Figure 6:**
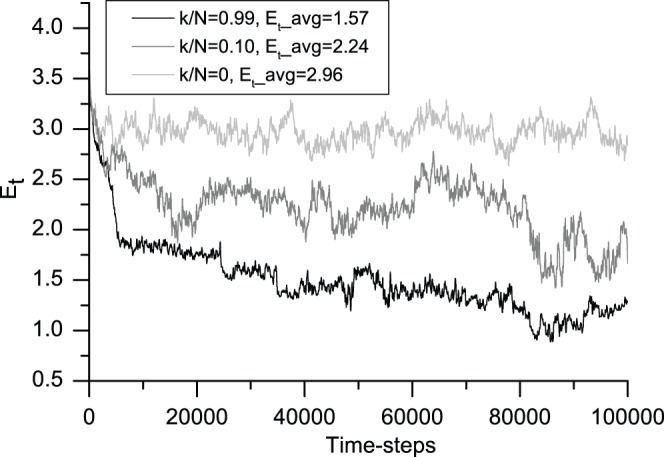
Evolution in simple social entropy associated with the degree of information sharing. This figure shows the 200-iteration moving average of social entropy. From top to bottom, light gray (*k/N* = 0), gray (*k/N* = 0.10) and black (*k/N* = 0.99) correspond to the typical cases from no information sharing to the most information sharing, respectively. The average simple social entropy values *E_t_avg_* for the three cases are 2.96, 2.24 and 1.57, respectively. This figure shows that in this model, no information sharing results in the least ordered system with the highest entropy, whereas the pheromone-like information sharing results in the most ordered system with the lowest entropy. All of the averages are calculated after the first 10,000 time-steps.

Entropy is popularly described as the degree of disorder in a system. Figure 6 shows that the simple social entropy associated with the information-sharing amount *k/N* = 0.99 clearly decreases over time. A similar but smaller decrease occurs with *k/N* = 0.10. For the no information-sharing mechanism, the simple social entropy remains almost constant (the light gray curve at the top of Figure 6). Figure 6 shows that the lowest degree of information sharing results in the least ordered system with the highest entropy, whereas the highest degree of information-sharing results in the most ordered system with the lowest entropy.

### 4 Successful Trading Ratio

**Figure 7:**
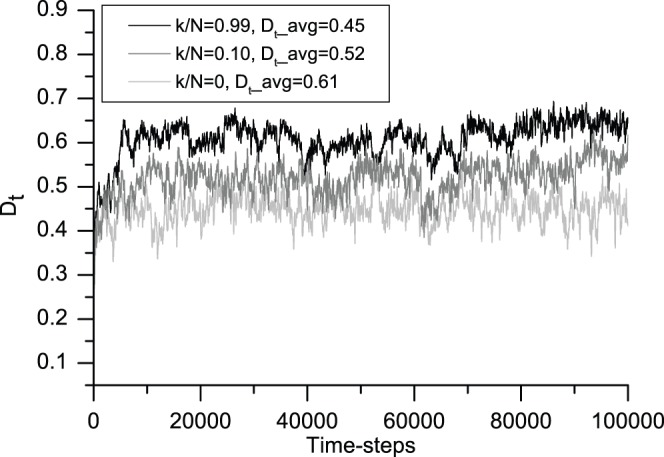
Evolution in the successful trade ratio associated with the degree of information sharing. This figure shows the 200-iteration moving average of the successful trade ratio. From bottom to top, light gray (*k/N* = 0), gray (*k/N* = 0.10) and black (*k/N* = 0.99) correspond to typical cases from no information sharing to the greatest degree of information sharing, respectively. The average successful trade ratios *D_t_avg_* associated with the three cases are 0.45, 0.52 and 0.61, respectively. In this model, more information promotes more successful trades. All of the averages are calculated after the first 10,000 time-steps.

Figure 7 shows that the successful trading ratio of the system changes over time. The three curves in Figure 7 represent the three cases for the degree of information sharing. Localized global information sharing generates the largest number of successful trades because more information is available with this mechanism, and each trader can find the best partner in a shorter time. We can conclude that the more information each agent can access, the larger the successful trading ratio.

### 5 Market Size Distribution

**Figure 8:**
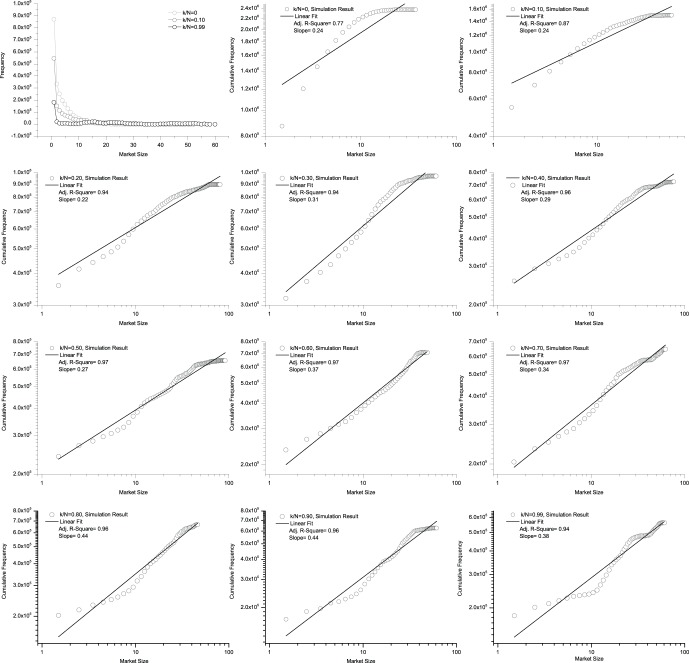
Market size distributions when the extent of information sharing varies from less to more. The three curves in the top left panel represent typical cases for information sharing, *k/N* = 0, 0.10, 0.99. To keep the curves clearly visible, we do not show all of the cases with *k/N* ranging from 0 to 0.99 in this panel. The rest of the panels in Figure 8 contain log-log plots of the cumulative distribution and a linear fit for all the cases *k/N* = 0, 0.10, 0.20, …, 0.90, 0.99. The market size frequency is calculated for 100,000 time-steps after the first 10,000 time steps. For *k/N* = 0, 0.10, the linear fits have an Adjusted R-Square less than 0.90, which means that there is not strong evidence of a power-law distribution. For *k/N*≥0.20, however, all of the linear fits have an Adjusted R-Square greater than 0.90, which means that there is strong evidence of a power-law distribution.

To further study the spatial structure of the market, we assess the market size when the system becomes stationary. Figure 8 shows the market size distributions after 100,000 time-steps. The three curves in the top left panel represent typical cases for the degree of information sharing, *k/N* = 0, 0.10, 0.99 (to keep the curves clearly visible, we do not show all of the cases with *k/N* ranging from 0 to 0.99 in this panel). The rest of the panels in Figure 8 contain log-log plots of the cumulative distribution and a linear fit for all the cases *k/N* = 0, 0.10, 0.20, …, 0.90, 0.99.

**Figure 9:**
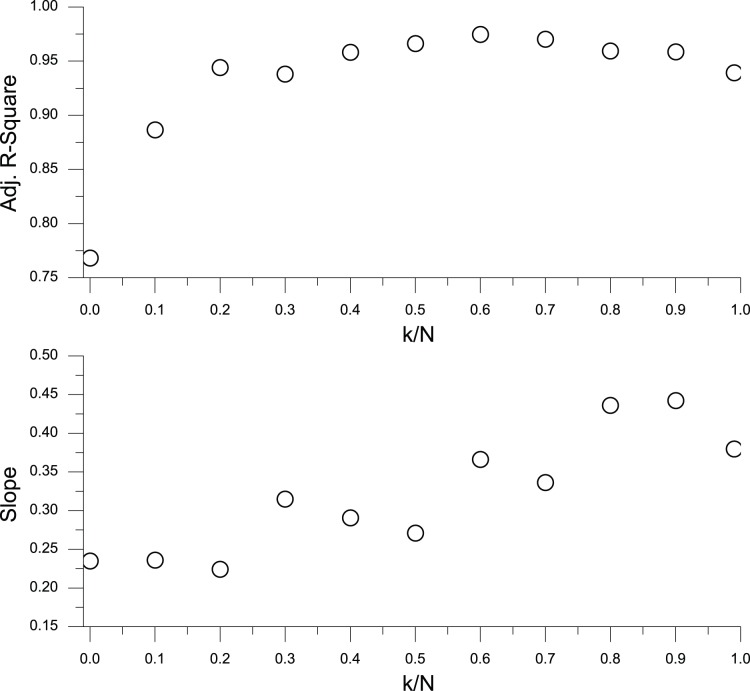
The Adjusted R-Square versus *k/N* and slope versus *k/N* for the linear fits in **Figure 8**.

If the frequency count *Q* versus the market size *Z* is a power-law distribution 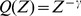, the cumulative distribution representing 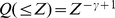
is also a power-law and vice versa. For *k/N* = 0, 0.10, the linear fits have an Adjusted R-Square of less than 0.90, which means that there is not strong evidence for a power-law distribution. For *k/N*≥0.20, however, all of the linear fits have an Adjusted R-Square greater than 0.90, which means strong evidence for a power-law distribution. Figure 9 is the Adjusted R-Square versus *k/N* and slope versus *k/N* for the linear fits in Figure 8. These two figures mean that with some information sharing, the power-law distribution of market size is a robust conclusion.

**Figure 10:**
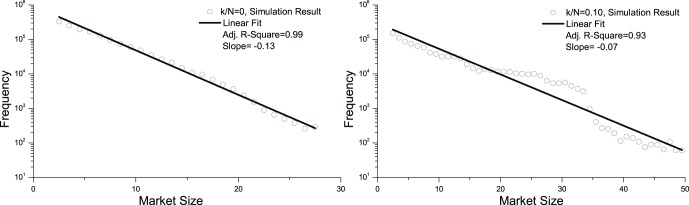
The linear fit in the log-linear scale for *k/N* = 0, 0.10. This figure shows the log-linear plot of market size distribution and linear fit for *k/N* = 0, 0.10. The linear fits have an Adjusted R-Square greater than 0.90, which means that the market size distributions for these two *k/N* ratios are exponential.

Figure 10 shows the log-linear plot of the market size distribution and the linear fit for *k/N* = 0, 0.10. The linear fits have an Adjusted R-Square greater than 0.90, which means that the market size distributions for these two *k/N* ratios are exponential.

**Figure 11:**
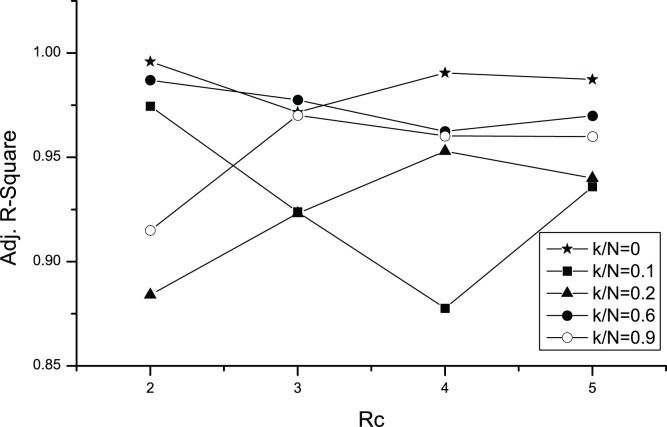
Sensitivity analysis of the Adjusted R-Square versus *R_c_* for some typical *k/N* values. There are only two dots (*k/N* = 0.10, *R_c_* = 4), (*k/N* = 0.20, *R_c_* = 2) that are below 0.90, which means that the results obtained from the distribution are quite robust to the variation of *R_c_* values, especially when there is no information sharing (*k/N* = 0) or the extent of information sharing is high (*k/N* = 0.60, 0.90).

Figure 11 shows the sensitivity analysis of the Adjusted R-Square versus *R_c_* ranging from 2 to 5 for some of the typical *k/N* values. Analysis for *R_c_* = 1 is omitted because it is unreasonable when sigh range *r = *2. There are only two dots, (*k/N* = 0.10, *R_c_* = 4) and (*k/N* = 0.20, *R_c_* = 2), that are below 0.90, which means that the results obtained for the distribution are quite robust to the variation of *R_c_* values, especially when there is no information sharing (*k/N* = 0) or when the extent of information sharing is high (*k/N* = 0.60, 0.90).

Power-law distributions have been observed in a diverse range of fields, including biology, economics, sociology, engineering and physics. Several well-known examples of phenomena that exhibit “scaling” behavior are city sizes [30], firm sizes [31], word frequencies [32], the frequencies of family names [33] and the sizes of earthquakes [34]. Certain mechanisms that involve growth and preferential attachment give rise to power-law distributions [35]. In this current study, when the agents can share more information, they tend to move to locations that are associated with more information. This process is similar to preferential attachment. Our results indicate that the larger size markets are more likely to form with pheromone-like information sharing.

### Conclusions

In this paper, we propose an agent-based market model that considers a spectrum of different information-sharing mechanisms. We also develop a unified model that accommodates all three of these information-sharing mechanisms based on a specific parameter–the number of friends per agent over the number of all agents, *k/N*.

The results of this study indicate that information plays a significant role in the agglomeration of economic activities (represented in this study as the spatial structure of a market). The amount of information shared gives rise to different market spatial structures. While no information sharing (*k/N* = 0) and little information sharing (*k/N* = 0.10) lead to exponential distributions, more information sharing (*k/N*≥0.20) results in a power-law distribution of market size; this result means that with some information sharing, the power-law distribution of market size is robust. The power-law distribution of market size corresponds to the stylized fact of city size and firm size distributions.

The breaking of symmetry [36] from an evenly distributed homogeneous initial state to a heterogeneous geographic structure is due to the tendency to select the previously traded sites based on the various information-sharing mechanisms (centrifugal forces) and the random walk (centripetal forces). This symmetry breaking process is a vivid example of B. Arthur’s viewpoint about the complexity research on what economists call path dependence, positive feedback and asymmetry [5]. Although the loci with higher levels of pheromone attract more traders, the cluster size does not increase without limit. Instead, the agents tend to form decentralized clusters.

This paper provides an alternative approach, agent-based experiments, which are the first that we know of in the economic geography field, to study the geographic aspects of economic development based on Krugman’s model. As far as we know, this is the first work that studies the contribution of only information sharing to the spatial structure of an economic system.

This model could be used as a test bed to validate assumptions such as the one made in this paper about which information-sharing mechanism is likely to regulate the real economic agglomeration. Furthermore, this study may elucidate the value of studying the functionality of information rather than counting information in bits and bytes.

In the Model section, we simplified the real-world economy to obtain a plausible model. We believe that this work can be extended by introducing the factors listed in the Model section. We predict that if this extension is performed, the information-sharing mechanism will be found to have a more complex effect on the spatial structure of the market at the global level.

## References

[1] Krugman P (1999) The role of geography in development. International regional science review 22: 142–161.

[2] Gallup JL, Sachs JD, Mellinger AD (1998) Geography and economic development.: National Bureau of Economic Research.

[3] Fujita M, Thisse JF (1996) Economics of agglomeration. Journal of the Japanese and international economies 10: 339–378.

[4] Ottaviano GIP, Puga D (1998) Agglomeration in the global economy: A survey of the ‘new economic geography’. The World Economy 21: 707–731.

[5] Arthur WB (1999) Complexity and the economy. Science 284: 107–109. 10103172 10.1126/science.284.5411.107

[6] Kirman A (2007) Course 5 economies with interacting agents. Les Houches 85: 217–255.

[7] Akerlof GA (1970) The market for “lemons”: Quality uncertainty and the market mechanism. The quarterly journal of economics: 488–500.

[8] Challet D, Zhang YC (1997) Emergence of cooperation and organization in an evolutionary game. Physica A: Statistical and Theoretical Physics 246: 407–418.

[9] Chli M, De Wilde P (2008) The emergence of knowledge exchange: an agent-based model of a software market. Systems, Man and Cybernetics, Part A: Systems and Humans, IEEE Transactions on 38: 1056–1067.

[10] Anand KS, Mendelson H (1997) Information and organization for horizontal multimarket coordination. Management Science: 1609–1627.

[11] Zhang YC (2005) Supply and demand law under limited information. Physica A: Statistical Mechanics and its Applications 350: 500–532.

[12] Gavirneni S, Kapuscinski R, Tayur S (1999) Value of information in capacitated supply chains. Management science: 16–24.

[13] Axtell RL, Epstein JM, Dean JS, Gumerman GJ, Swedlund AC, et al (2002) Population Growth and Collapse in a Multiagent Model of the Kayenta Anasazi in Long House Valley. Proceedings of the National Academy of Sciences of the United States of America 99: 7275–7279. 12011406 10.1073/pnas.092080799 PMC128597

[14] Schelling TC (1971) Dynamic models of segregation†. Journal of mathematical sociology 1: 143–186.

[15] Lim M, Metzler R, Bar-Yam Y (2007) Global pattern formation and ethnic/cultural violence. Science 317: 1540–1544. 17872443 10.1126/science.1142734

[16] Macy MW, Sato Y (2002) Trust, cooperation, and market formation in the US and Japan. Proceedings of the National Academy of Sciences of the United States of America 99: 7214. 12011400 10.1073/pnas.082097399 PMC128588

[17] Sabater J, Sierra C (2005) Review on computational trust and reputation models. Artificial Intelligence Review 24: 33–60.

[18] Kohonen T (1990) The self-organizing map. Proceedings of the IEEE 78: 1464–1480.

[19] Epstein JM, Axtell R (1996) Growing artificial societies: social science from the bottom up: The MIT Press.

[20] Lim M, Metzler R, Bar-Yam Y (2007) Global pattern formation and ethnic/cultural violence. Science 317: 1540. 17872443 10.1126/science.1142734

[21] Dorigo M, Stützle T (2004) Ant Colony Optimization.: The MIT Press.

[22] Bonabeau E, Sobkowski A, Theraulaz G, Deneubourg JL (1997) Adaptive task allocation inspired by a model of division of labor in social insects. Biocomputing and Emergent Computation: 36–45.

[23] Zhang YC (2005) Supply and demand law under limited information. Physica A: Statistical Mechanics and its Applications 350: 500–532.

[24] Sumpter DJT, Pratt SC (2009) Quorum responses and consensus decision making. Philosophical Transactions of the Royal Society B: Biological Sciences 364: 743–753. 10.1098/rstb.2008.0204 PMC2689713 19073480

[25] Ester M, Kriegel HP, Sander J, Xu X (1996) A density-based algorithm for discovering clusters in large spatial databases with noise. AAAI Press. 226–231.

[26] Ioannou CC, Guttal V, Couzin ID (2012) Predatory Fish Select for Coordinated Collective Motion in Virtual Prey. Science 337: 1212–1215. 22903520 10.1126/science.1218919

[27] Gorelick R, Bertram SM (2007) Quantifying division of labor: borrowing tools from sociology, sociobiology, information theory, landscape ecology, and biogeography. Insectes sociaux 54: 105–112.

[28] Balch T (2000) Hierarchic social entropy: An information theoretic measure of robot group diversity. Autonomous robots 8: 209–238.

[29] Dorigo M, Blum C (2005) Ant colony optimization theory: A survey. Theoretical computer science 344: 243–278.

[30] Gabaix X (1999) Zipf’s law for cities: an explanation. The Quarterly Journal of Economics 114: 739–767.

[31] Axtell RL (2001) Zipf distribution of US firm sizes. Science 293: 1818–1820. 11546870 10.1126/science.1062081

[32] Zipf GK (1929) Relative frequency as a determinant of phonetic change. Harvard Studies in Classical Philology 40: 1–95.

[33] Zanette DH, Manrubia SC (2001) Vertical transmission of culture and the distribution of family names. Physica A: Statistical Mechanics and its Applications 295: 1–8.

[34] Gutenberg B, Richter CF (1944) Frequency of earthquakes in California. Bulletin of the Seismological Society of America 34: 185–188.

[35] Albert R, Barabási AL (2002) Statistical mechanics of complex networks. Reviews of modern physics 74: 47.

[36] Anderson PW (1972) More is different. Science 177: 393–396. 17796623 10.1126/science.177.4047.393

